# Supplementary material for: How quickly can we predict trimethoprim resistance using alchemical free energy methods?
Source: Interface Focus. 2020 Oct 16;10(6):20190141. doi: 10.1098/rsfs.2019.0141 (PMC7653339; doi:10.1098/rsfs.2019.0141)
Supplement: Supplemental Figures and Table [file rsfs20190141supp1.pdf]

How quickly can we predict trimethoprim resistance using alchemical free energy methods? – Supplemental Material[*]

Philip W Fowler[†1,2]

[1]Nuffield Department of Medicine, John Radcliffe Hospital, University of Oxford, Headley Way, Oxford, OX3 9DU, UK

[2]National Institute of Health Research Oxford Biomedical Research Centre, John Radcliffe Hospital, Headley Way, Oxford, OX3 9DU, UK

Tables of Free Energies

Three data tables – one for Set1, one for Set2 and one for Set 3 (Table 1) – are included along with the paper. These were produced by analysing the alchemical molecular dynamics simulations and were used in all the analyses in the main body of the paper, as well as the figures in this Supplement. Each table has free energies calculated at 500 different values of the simulation duration, t, and hence Δt is 0.5ps, 5 ps and 50 ps for Set1, Set2 and Set3, respectively. Each calculation is uniquely identified by the combination of `CALCULATION` and `RUN`. `CALCULATION` only takes one of three values `ti-02`, `ti-05` or `ti-12`. Those labelled `ti-05` contains 10 different values of `RUN` and these are the calculations which were extended from 0.25 ns to 2.5 ns and therefore make up Set2, whilst the `ti-12` contains five separate calculations for the F99Y mutation generated from simulations 25 ns long. Hence, with the exception of F99Y, `dhfr-Set2.csv` only contains `CALCULATION=='ti-05'` whilst `dhfr-Set1.csv` contains both values. The tables not only contain $\Delta\Delta G_{tmp}$ (labelled `ddG_tmp`), but also values of $\Delta\Delta G_{fol}$ (labelled `ddG_fol`, if available) and the individual alchemical free energies used to calculate each. These are labelled with a numerical subscript which refers to the thermodynamic cycle found in Fig. S1. Note that Fig. S1 only contains the labels for $\Delta\Delta G_{tmp}$: those for $\Delta\Delta G_{fol}$ map as follows: `ddG_5` $\rightarrow$ `ddG_8`, `ddG_6[1,2,3]` $\rightarrow$ `ddG_9[1,2,3]` and `ddG_7` $\rightarrow$ `ddG_10`.

*published in the Royal Society journal, *Interface Focus*

†To whom correspondence should be addressed: philip.fowler@ndm.ox.ac.uk, @philipwfowler

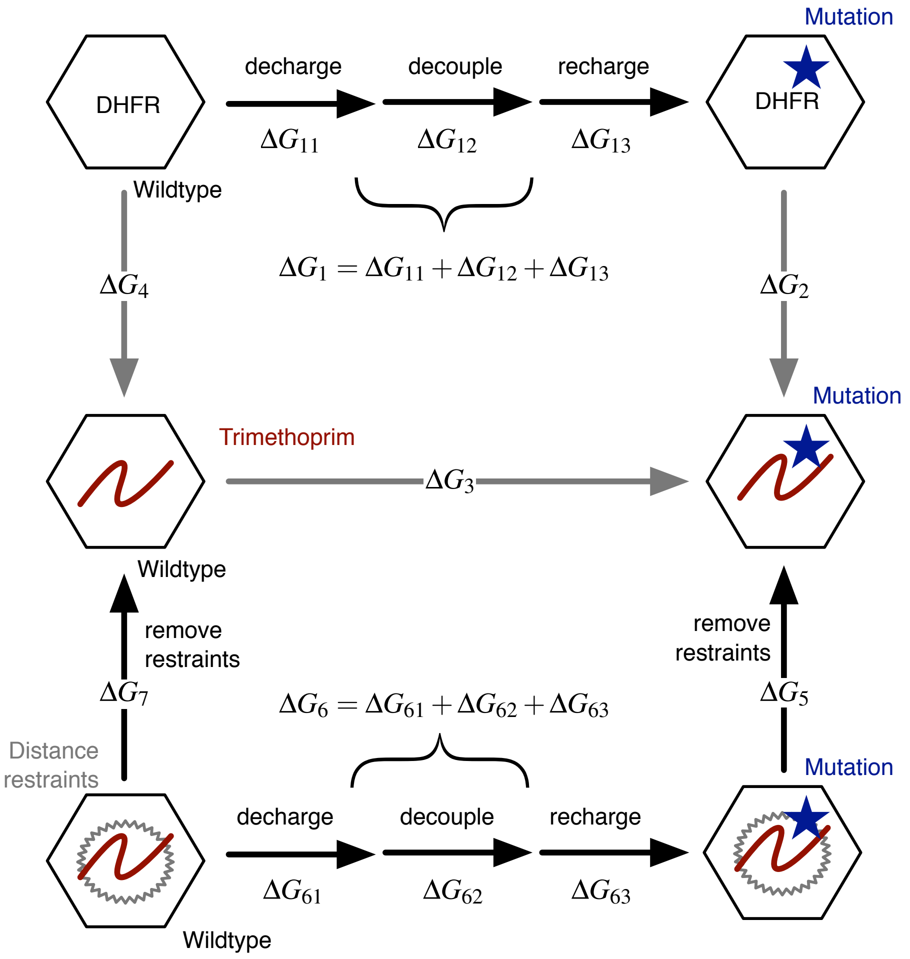

Figure S1: (Related to Equation 1) The thermodynamic cycle used to calculate $\Delta\Delta G_{tmp}$. The nomenclature is used throughout the main body as well as in the included data tables.

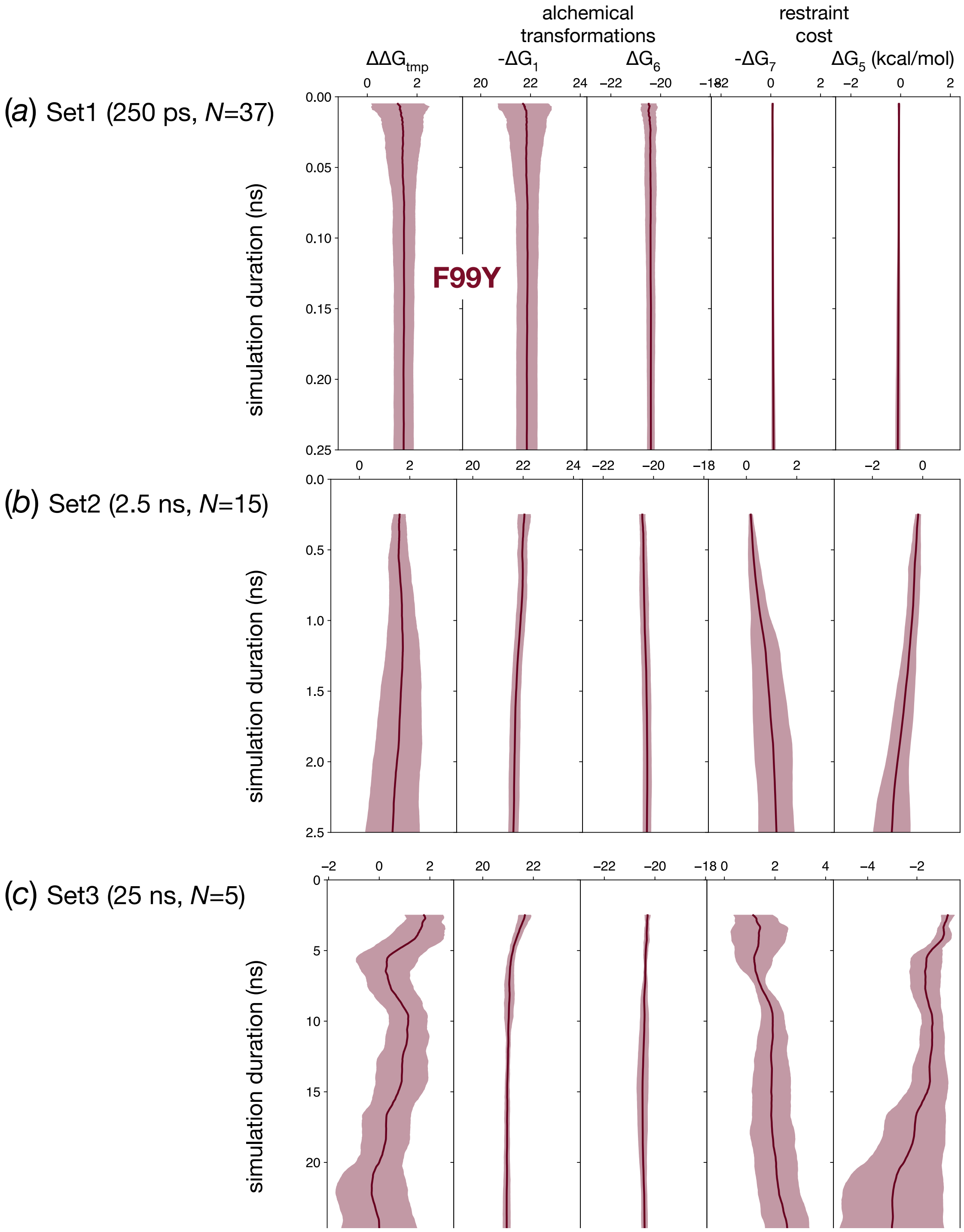

Figure S2: (Related to Figure 2) The mean and variance of the four free energies used to calculate $\Delta\Delta G_{tmp}$ for the F99Y mutation as described by the thermodynamic cycle in Fig. S1 using the varying numbers of simulations belonging to Sets 1, 2 & 3. To aid comparison, the x-axis of each graph covers the same magnitude of energy (5 kcal/mol) and the resulting value of $\Delta\Delta G_{tmp}$ is also shown.

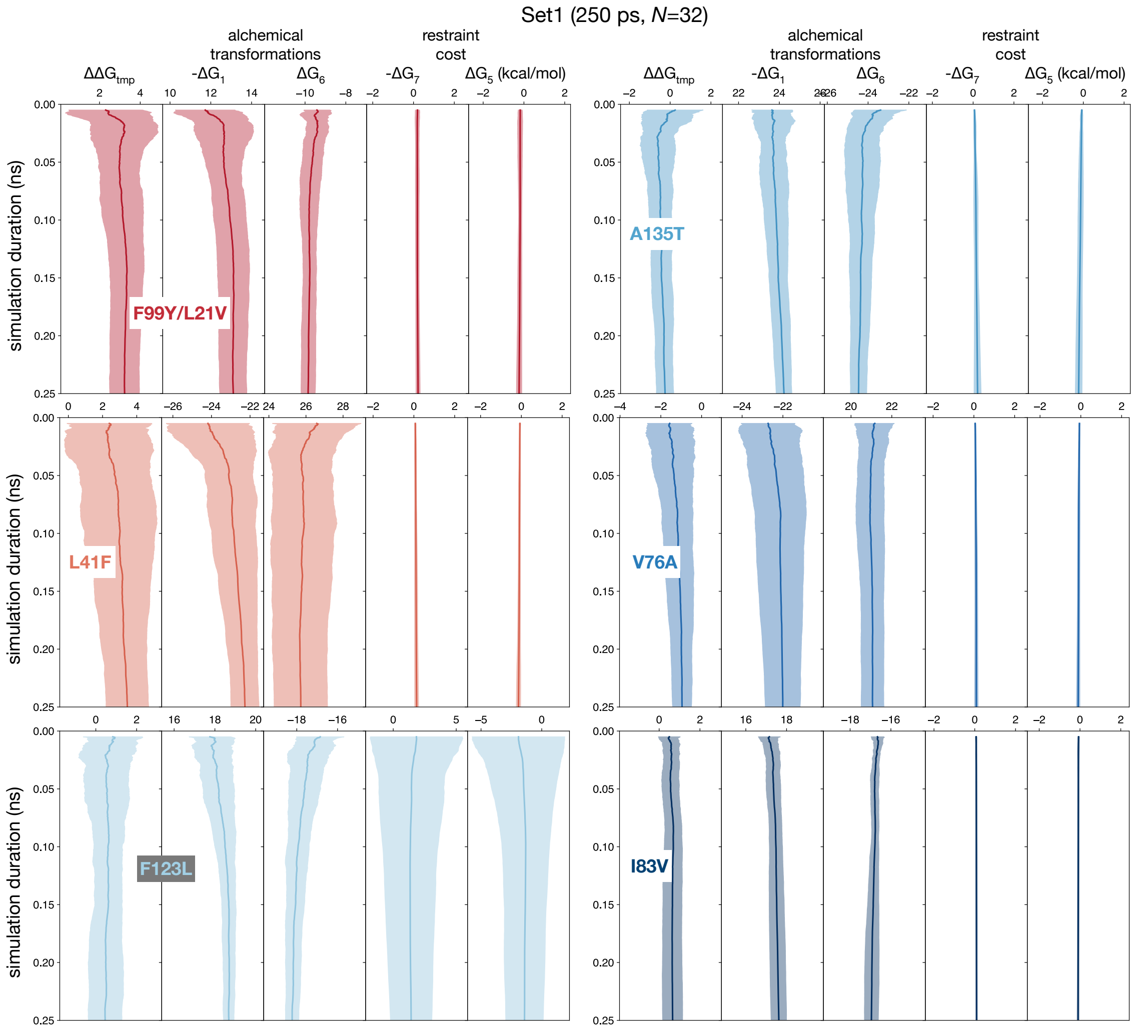

Figure S3: (Related to Figure 3) The mean and variance of the four free energies used to calculate $\Delta\Delta G_{tmp}$ for the other six mutations as described by the thermodynamic cycle in Fig. S1 using the 32 simulations belonging to Set1. To aid comparison, the x-axis of each graph covers the same magnitude of energy (5 kcal/mol) and the resulting value of $\Delta\Delta G_{tmp}$ is also shown.

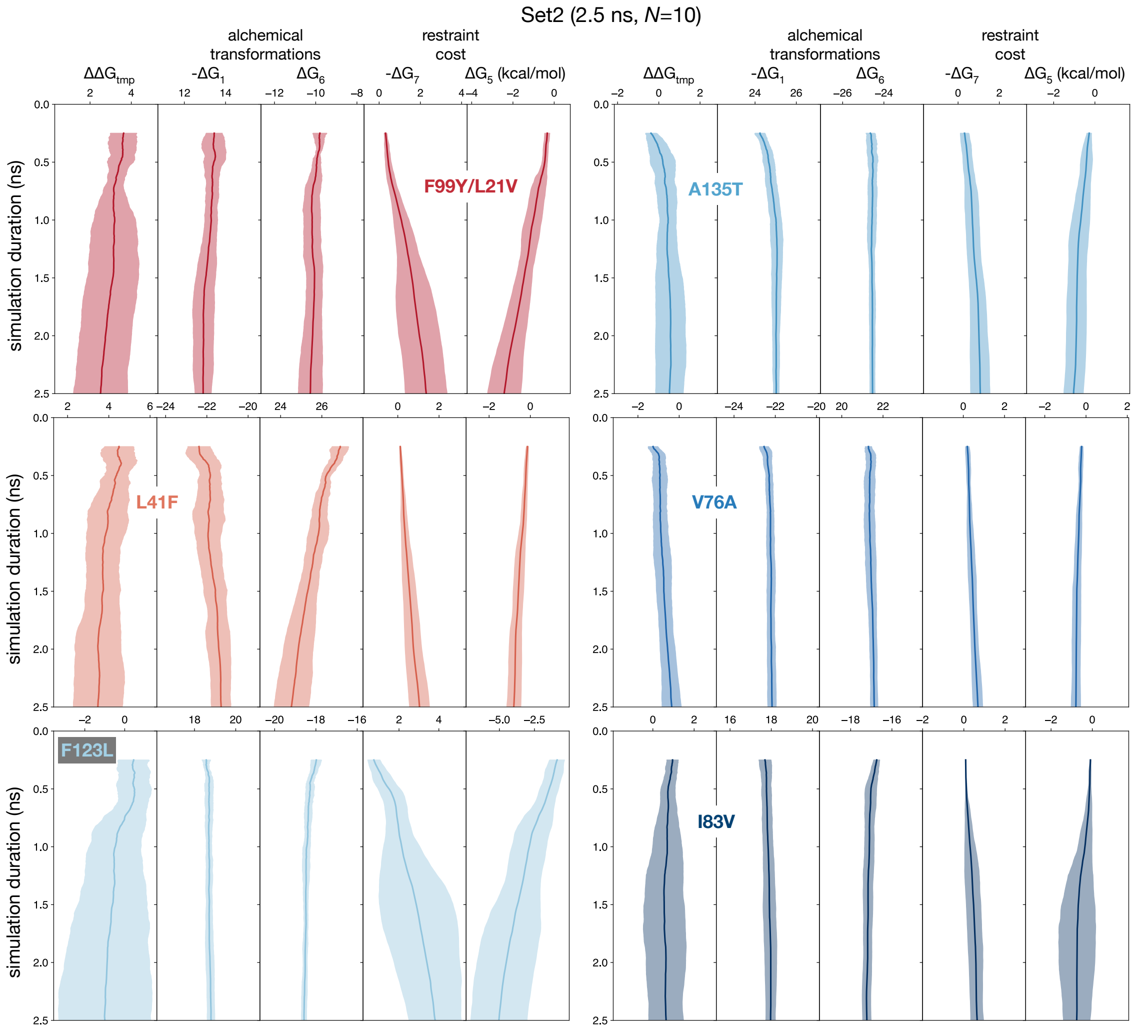

Figure S4: (Related to Figure 3) The mean and variance of the four free energies used to calculate $\Delta\Delta G_{tmp}$ for the other six mutations as described by the thermodynamic cycle in Fig. S1 using the 10 simulations belonging to Set2. To aid comparison, the x-axis of each graph covers the same magnitude of energy (5 kcal/mol) and the resulting value of $\Delta\Delta G_{tmp}$ is also shown.

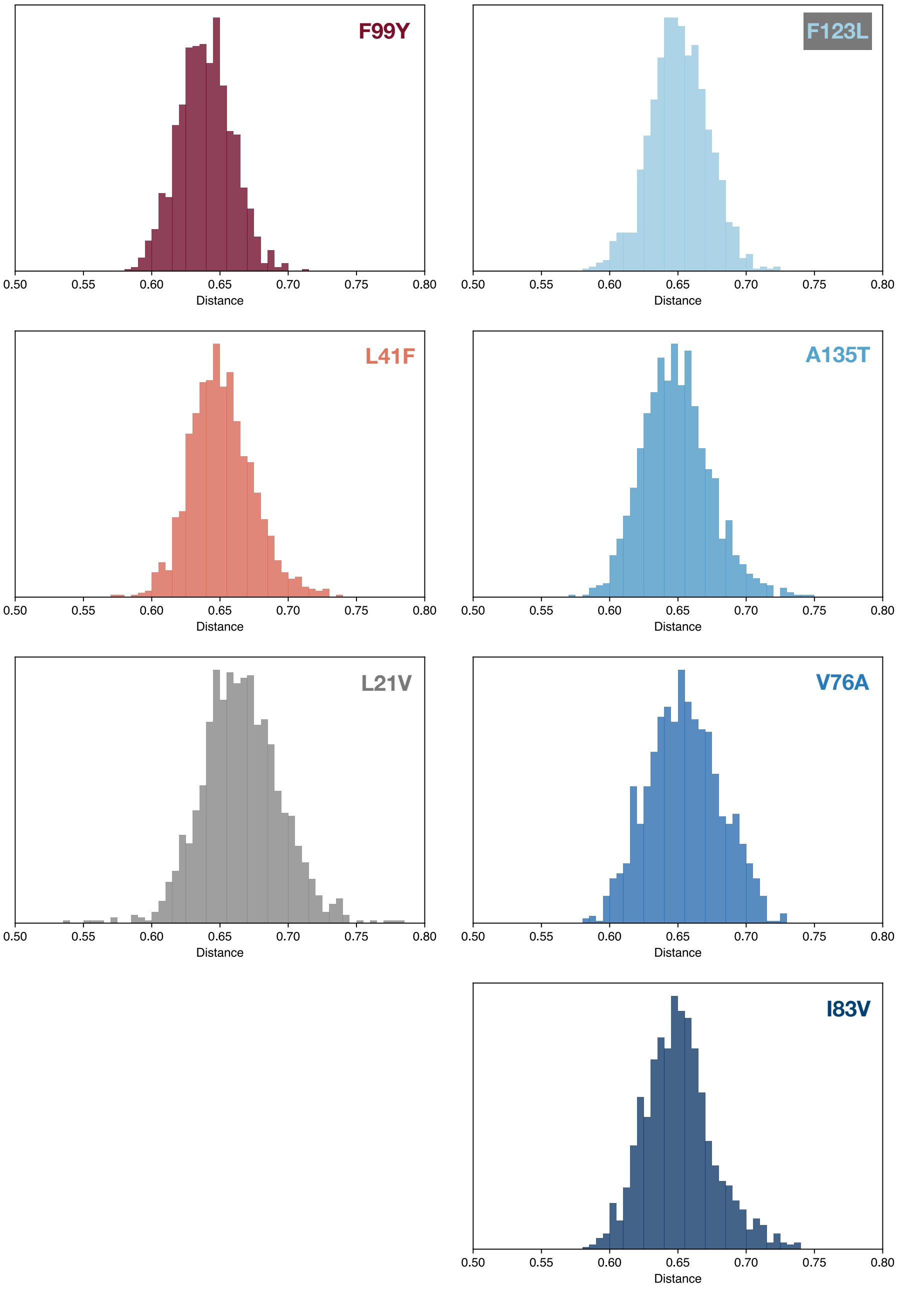

Figure S5: The ligand (trimethoprim) remained bound to the protein in 5× 25 ns unrestrained simulations, suggesting that restraints are not required for these mutations. The distance between the centre of mass of the protein and ligand was calculated for each simulation; these were aggregated for each mutation and a histogram plotted. Note that the Y99L21V simulation was not considered, hence L21V (grey) is shown.

Table S1: Five of the seven amino acid mutations studied were found in a search index of microbial genomes deposited into the European Nucleotide Archive. Only sets of short reads that were assessed as containing at least 80% *S. aureus* were included. Since the index is queried using a *k*-mer, rather than the whole gene, the total number of genomes detected varied across the *dfrB* gene with an average value of 19,200.

Position	Reference	Mutation	# Genomes	New triplet
12	caa	Q12R	9	cga
14	gta	V14A	4	gca
		V14I	1	ata
18	gaa	E18K	3	aaa
21	tta	L21I	2	ata
		L21V	421	gta
25	cta	L25I	3	ata
31	cat	H31N	15	aat
		H31Y	6	tat
34	aaa	K34E	8	gaa
35	tta	L35S	8	tca
		L35I	10	ata
41	tta	L41F	1	ttt
		L41F	2	ttc
43	atg	M43L	4	ttg
44	ggt	G44A	3	gct
54	cca	P54S	33	tca
		P54L	1	cta
		P54Q	1	caa
56	ccg	P56L	1	ctg
		P56S	12	tcg
		P56A	1	gcg
60	aat	N60I	408	att
62	gta	V62A	3	gca
		V62L	6	tta
67	aca	T67I	2	ata
		T67S	2	tca
		T67K	4	aaa
71	gta	V71I	12	ata

Continued on next page

Table S1: Five of the seven amino acid mutations studied were found in a search index of microbial genomes deposited into the European Nucleotide Archive. Only sets of short reads that were assessed as containing at least 80% *S. aureus* were included. Since the index is queried using a *k*-mer, rather than the whole gene, the total number of genomes detected varied across the *dfrB* gene with an average value of 19,200.

Position	Reference	Mutation	# Genomes	New triplet
73	ggc	G73D	2357	gac
		G73S	1	agc
76	gta	V76A	1412	gca
		V76L	1	tta
78	cat	H78R	2	cgc
		H78P	1	ccc
		H78Y	1	tat
99	ttt	F99Y	137	tat
103	att	I103V	8	gtt
105	aaa	K105R	4	aga
106	gtg	V106A	1	gcg
		V106M	7	atg
		V106E	1	gag
125	cca	P125Q	4	caa
		P125L	2	cta
131	gac	D131G	1	ggc
		D131N	1	aac
		D131Y	1	tac
134	gtt	V134I	823	att
135	gcc	A135T	6578	acc
		A135S	1	tcc
		A135I	1	atc
140	ggt	G140C	9	tgt
144	gag	E144G	9	gga
		E144D	3	gat
		E144G	5	ggg
